# Hydroxyethyl Starch (HES 130/0.4) Impairs Intestinal Barrier Integrity and Metabolic Function: Findings from a Mouse Model of the Isolated Perfused Small Intestine

**DOI:** 10.1371/journal.pone.0121497

**Published:** 2015-03-23

**Authors:** Yuk Lung Wong, Ingmar Lautenschläger, Heike Dombrowsky, Karina Zitta, Berthold Bein, Thorsten Krause, Torsten Goldmann, Inez Frerichs, Markus Steinfath, Norbert Weiler, Martin Albrecht

**Affiliations:** 1 Department of Anesthesiology and Intensive Care Medicine, University Medical Center Schleswig-Holstein, Kiel, Germany; 2 Division of Barrier Integrity, Research Center Borstel, Leibniz-Center for Medicine and Biosciences, Borstel, Germany; 3 Division of Mucosal Immunology and Diagnostics, Research Center Borstel, Leibniz-Center for Medicine and Biosciences, Borstel, Germany; 4 Division of Clinical and Experimental Pathology, Research Center Borstel, Leibniz-Center for Medicine and Biosciences, Borstel, Germany; University of Florida, UNITED STATES

## Abstract

**Background:**

The application of hydroxyethyl starch (HES) for volume resuscitation is controversially discussed and clinical studies have suggested adverse effects of HES substitution, leading to increased patient mortality. Although, the intestine is of high clinical relevance and plays a crucial role in sepsis and inflammation, information about the effects of HES on intestinal function and barrier integrity is very scarce. We therefore evaluated the effects of clinically relevant concentrations of HES on intestinal function and barrier integrity employing an isolated perfused model of the mouse small intestine.

**Methods:**

An isolated perfused model of the mouse small intestine was established and intestines were vascularly perfused with a modified Krebs-Henseleit buffer containing 3% Albumin (N=7) or 3% HES (130/0.4; N=7). Intestinal metabolic function (galactose uptake, lactate-to-pyruvate ratio), edema formation (wet-to-dry weight ratio), morphology (histological and electron microscopical analysis), fluid shifts within the vascular, lymphatic and luminal compartments, as well as endothelial and epithelial barrier permeability (FITC-dextran translocation) were evaluated in both groups.

**Results:**

Compared to the Albumin group, HES perfusion did not significantly change the wet-to-dry weight ratio and lactate-to-pyruvate ratio. However, perfusing the small intestine with 3% HES resulted in a significant loss of vascular fluid (p<0.01), an increased fluid accumulation in the intestinal lumen (p<0.001), an enhanced translocation of FITC-dextran from the vascular to the luminal compartment (p<0.001) and a significantly impaired intestinal galactose uptake (p<0.001). Morphologically, these findings were associated with an aggregation of intracellular vacuoles within the intestinal epithelial cells and enlarged intercellular spaces.

**Conclusion:**

A vascular perfusion with 3% HES impairs the endothelial and epithelial barrier integrity as well as metabolic function of the small intestine.

## Introduction

A common therapy for the treatment of hypovolemia is the application of crystalloid and colloidal solutions for fluid resuscitation [1]. Among others, hydroxyethyl starch (HES), a synthetic nonionic starch derivate which is available in various molecular weight and substitution forms, is frequently applied in the clinic [1, 2]. However, several recently published studies have suggested a negative benefit-risk ratio of HES, showing an increased mortality after fluid resuscitation with HES [1, 3, 4].

The intestine is a typical barrier organ with a large inner surface area and one of its major function is to maintain a selective barrier between the organism and the environment [5]. Under physiological conditions the intestinal endo- and epithelia preserve the fluid homeostasis and barrier function between the vascular, interstitial and luminal compartments. This important function is for example impaired during inflammatory processes and microbial sepsis, which induce an increased endothelial and epithelial permeability leading to intestinal edema formation and passage of bacterial toxins as well as pathogens into the systemic circulation [6–10]. In spite of the central role of the intestine in metabolism, inflammation and sepsis, information about the effects of HES solutions on intestinal function and barrier integrity is still very scarce [8, 11, 12].

To gain insight into the possible effects of HES on intestinal function and barrier integrity, we evaluated the HES mediated cellular effects employing a newly established isolated perfused model of the mouse small intestine showing that the vascular perfusion with clinically relevant concentrations of HES impairs the endothelial and epithelial barrier integrity as well as metabolic function of the intestine.

## Materials and Methods

### Animals

Female C57/BL6 mice (15–25g; Charles River, Sulzfeld, Germany) were used for all experiments. Animals were housed with standard diet and water *ad libitum* for at least 24 hours before surgery. This study was carried out in accordance with the recommendations in the Guide for the Care and Use of Laboratory Animals of the National Institutes of Health. The experiments were approved by the local authority (Ministry of Agriculture, Environment and Rural Areas of the State of Schleswig-Holstein, Kiel, Germany).

### Experimental protocol

**Fig 1:**
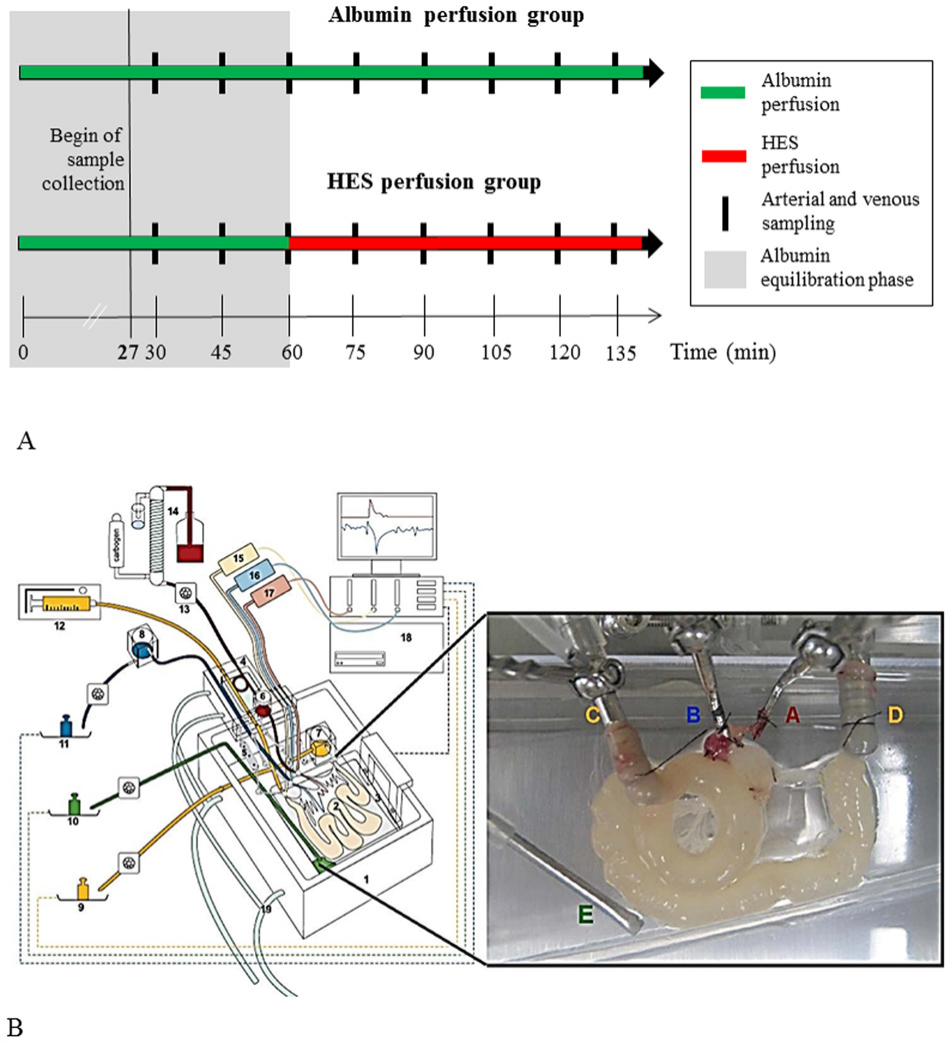
Experimental setting and basic components of the perfusion model. A) Experimental setting and time frame B) isolated perfused mouse small intestine apparatus. Using a custom made, heated chamber (1) an isolated small intestine (2) is perfused (vascular system: red, blue; luminal system: yellow; lymphatic system: green) while placed on a built-in microbalance (3). A moveable cannulating block (4) carries the tubings, heat exchanger cannula holders (5), and bubble trap (6). Height-adjustable reservoirs (7, 8) allow clamping both afterloads to zero. For online analysis of fluid homeostasis all emanating liquids are quantified by use of three balances (9 to 11). Constant flow perfusion is applied by a syringe pump (12) and a roller pump (13). The vascular perfusate is pH equilibrated, oxygenated and prewarmed with a tempered hollow fiber dialyzer flushed with carbogen gas (14). Pressure transducers (15–17) allow online detection of the luminal (yellow), venous (blue) and arterial (red) pressures. All data are recorded on a personal computer (18). To secure constant temperature, the chamber and cannulating block are water-jacketed and warmed by a water bath (19). The inset shows a representative photograph of a perfused small intestine. A, arterial cannula; B, venous cannula; C, oral intestinal lumen cannula; D, aboral intestinal lumen cannula; E, lymphatic suction needle; modified from [14]. A representative video presentation of the perfused small intestine is available as online supporting information (S1 Movie).

To evaluate the intestinal effects of a vascularly perfused HES solution, two experimental settings were established. The first group (“Albumin perfusion group”, control, N = 7) received a 135 min vascular perfusion with Albumin (3%) containing buffer. The second group (“HES perfusion group”, HES, N = 7) received a vascular Albumin (3%) perfusion for 60 min (equilibration phase) followed by a HES 130/0.4 (3%; Fresenius Kabi, Bad Homburg, Germany) perfusion for 75 min. Perfusions were continuously applied without intermittent stops. When establishing the mouse model of the isolated perfused intestine, various control experiments were performed to investigate the physiological and metabolic stability of the perfused intestines during the ex-vivo experiment. Employing the described experimental setup, mouse intestines are physiologically and metabolically stable for up to 135 minutes. This was the main reason, why all experiments were performed for a maximum time period of 135 minutes including an equilibration phase of 60 minutes. To exclude influences of the perfusion time on the observed effects, the results obtained at a respective time point in the HES perfusion group were compared to the respective time point in the Albumin perfusion group (inter group comparison) as well as to the “internal” HES control which consisted of 60 min equilibration with Albumin prior to the HES perfusion (intra group comparison; Fig. 1A).

### Preparation of the small intestine, cannulation and perfusion

Mice were anesthetized by inhalation of 1–3% sevoflurane and an additional intraperitoneal injection of ketamine up to a maximum dose of 40 mg/kg. After opening the abdomen by a midline incision, further preparation steps were performed under a binocular microscope. Parts of the duodenum, the jejunum and ileum were isolated for the perfusion as described in detail for the rat [13]. All animals were killed under narcosis by cervical dislocation. The cannulation system used for perfusing the mouse intestine was based on our recently published rat model [14]. Minor modifications and adaptions were necessary due to animal size and anatomical differences between the rat and mouse system (Fig. 1B). Briefly, we used a custom made perfusion system from Hugo Sachs Elektronik-Harvard Apparatus (March-Hugstetten, Germany) with modified cannulas and weight sensors. For perfusion, the aorta (in close proximity to the superior mesenteric artery), the hepatic portal vein as well as the proximal and distal small intestine were cannulated (see inset in Fig. 1B). For the vascular perfusion a modified Krebs-Henseleit solution containing 2 mM lactobionic acid, 7.4 mM glucose, 30 mM mannitol, 0.8 mM glutamine, 122 μg/l norepinephrine hydrochloride, 12.6 mM HEPES, and 3% Albumin (BSA, Sigma-Aldrich, Munich, Germany) was used for the equilibration period in the HES perfusion group (t = 0 min to t = 60 min) and the Albumin perfusion group (t = 0 min to t = 135 min). In the HES perfusion group Albumin was replaced after 60 min by 3% HES 130/0.4 (Fresenius Kabi, Bad Homburg, Germany). Both vascular perfusates had an adjusted pH of 7.4 and an osmolality of 300–325 mosmol/l. Perfusion of the small intestine was performed as described by Lautenschläger et al. [14] with minor modifications. Briefly, the single-pass perfusion via Tygon tubes and roller pump transported the vascular buffer for oxygenation through a dialyzer (FX paed, Fresenius Medical Care, Bad Homburg, Germany) with a rate of 2 ml/min. Constant luminal flow perfusion was applied by a syringe pump set to 0.06 ml/min. Vascular and luminal flow was continuously applied throughout the experiment without intermittent stops. The venous, luminal and lymphatic effluent weights were determined by high sensitive weight units connected to an online monitoring system (Hugo Sachs Elektronik-Harvard Apparatus, March-Hugstetten, Germany). A height adjustable reservoir for the venous and luminal outflow enabled sampling and reduction of afterload to prevent tissue damage and edema formation. The venous, lymphatic and luminal effluent volumes as well as the arterial, venous, and luminal perfusion pressures were recorded continuously. A blood gas analyser (ABL700, Radiometer Copenhagen, Bønshøj, Denmark) was used to measure O_2_ and CO_2_ partial pressures, pH, electrolytes, glucose and lactate of the arterial inflow and venous outflow every 15 minutes (Fig. 1B). A representative perfused small intestine with typical peristaltic movements can be seen in the supporting information provided online (S1 Movie).

### Pre- and post- perfusion tissue preparation

One 3 cm long proximal portion of the small intestine was obtained before as well as after perfusion and the mesentery was removed. The wet weight was determined immediately after depletion of intestinal liquid while for the evaluation of the dry weight, the same sample was dehumidified for 96 h at 55°C. Moreover, at the end of the perfusion experiment an approximately 3 cm long portion of the intestine was fixed in 4% formaldehyde for histological examination.

### Evaluation of the vascular lactate-to-pyruvate ratio

The lactate-to-pyruvate ratio was employed as a parameter for anaerobic and aerobic metabolism. Pyruvate was determined in the venous outflow by a quantitative enzymatic photometric method (Sigma-Aldrich, Munich, Germany). Additionally, the vascular lactate concentration was evaluated using a blood gas analyser (ABL700, Radiometer Copenhagen, Bønshøj, Denmark) and the lactate-to-pyruvate ratio was calculated.

### Quantification of vascular, lymphatic and luminal FITC-dextran

FITC-dextran (150 kDa; Sigma-Aldrich, Munich, Germany) was added into the vascular perfusate in a concentration of 40 mg/l to determine the endothelial and epithelial permeability for macromolecules. Under physiological conditions the endothelial and also the epithelial barrier is impermeable for the 150 kDa FITC-dextran [15]. Samples of venous, lymphatic, and luminal outflow were collected every 15 minutes and analysed for the FITC-dextran content using a fluorescence ELISA reader (excitation 485 nm, emission 530 nm; FL 600 microplate fluorescence Reader, MWG-Biotech, Ebersberg, Germany).

### Determination of vascular galactose

In order to evaluate the resorptive capacity of the intestine, 30 mM of lactose were supplied with the luminal perfusion buffer and vascular galactose (derived from the luminal lactose) was determined by a commercially available assay kit (Raffinose/D-Galactose Assay Kit, Megazyme, Bray, Ireland). Due to initially high variations in galactose uptake during the equilibration phase, statistical analyses were only performed with samples from time points 60 min and beyond.

### Histological examination

Sections of intestinal tissue were stained with hematoxylin-eosin and periodic acid-Schiff [16]. Analyses of tissue damage were performed by a blinded investigator employing a histological stability score described elsewhere with the exception that only longitudinal slices were included [14]. Tissue samples for transmission electron microscopy were prepared as described previously [17] and examined using an electron microscope (Zeiss E910, Jena, Germany).

### Statistical analysis

Statistical analyses were performed using GraphPad Prism 5 (GraphPad Software, San Diego, USA). Data are presented as mean values with standard deviations (SD). Statistical comparisons were performed using Student’s t-tests, one-way ANOVA (for intra group comparisons) and two-way ANOVA (for inter group comparisons) with Bonferroni post-tests. Differences were considered to be statistically significant if p was less than 0.05. Non-parametric data were analysed by Kruskal-Wallis test and Dunns post-test. For the two-way ANOVA data were transformed using [Y = log(Y)].

## Results

### Quantification of the vascular lactate-to-pyruvate ratio

**Fig 2:**
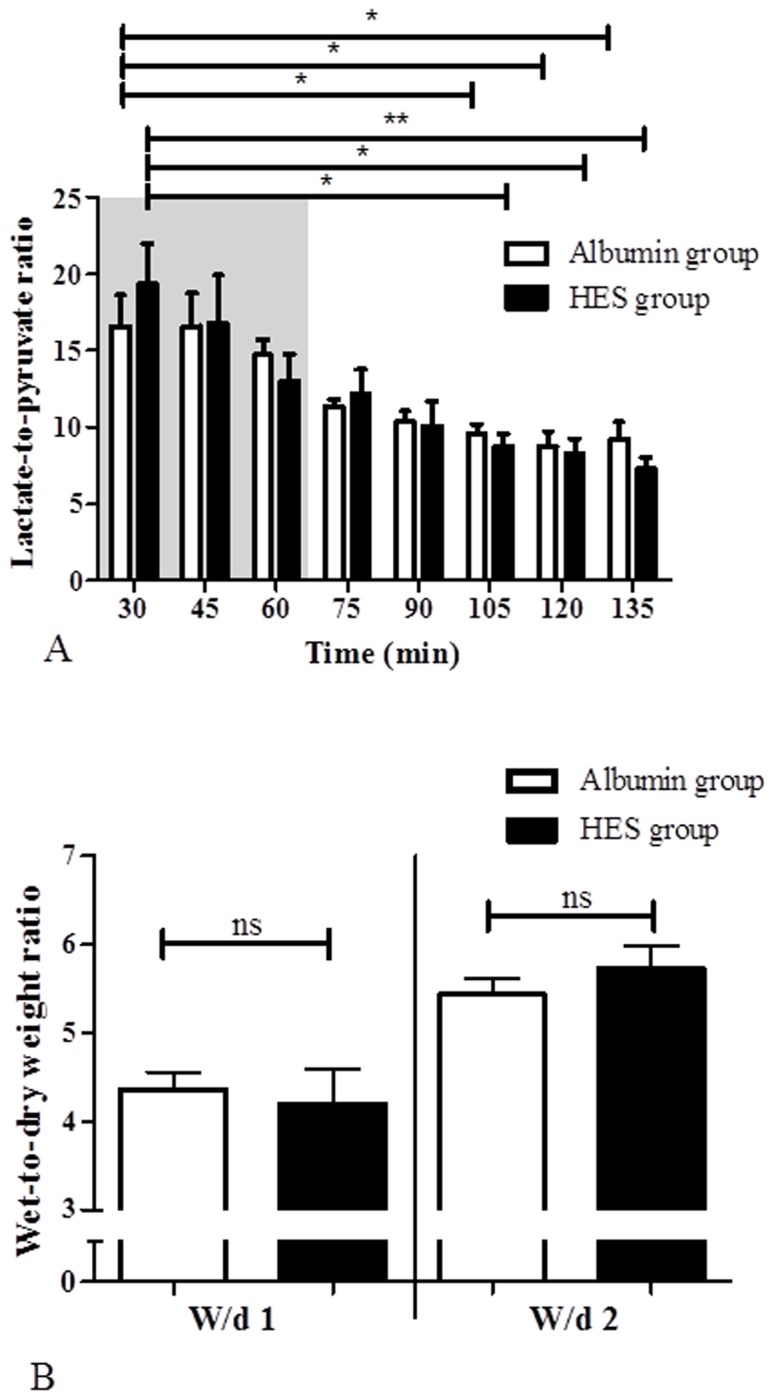
Lactate-to-pyruvate ratio and wet-to-dry weight ratio. A) Lactate-to-pyruvate ratio in the Albumin perfusion and HES perfusion group, B) wet-to-dry weight ratio before (W/d1) and after (W/d2) perfusion, in the Albumin perfusion and HES perfusion group. Areas shaded in grey indicate the equilibration phase. Error bars denote the mean ± SD. Albumin (N = 6), HES (N = 7). *, p < 0.05; **, p < 0.01; ns, not significant.

As a parameter for the metabolic status the vascular lactate-to-pyruvate ratio (LPR) was determined and all values were within the physiological range of aerobic metabolism. There were no statistically significant differences between the time response characteristics of the LPR in the HES perfusion group and the time response characteristics of LPR in the Albumin perfusion group (p > 0.05; inter group comparison; Fig. 2A). However, due to the equilibration of the system, a time dependent reduction of LPR was observed in the HES and Albumin perfusion group [HES _t30_ (19.36 ± 6.94) vs. HES _t105_ (8.74 ± 2.21); HES _t120_ (8.23 ± 2.75); HES _t135_ (7.28 ± 1.99); p < 0.05 for HES _t105_ and HES _t120_, p < 0.01 for HES _t135_; intra group comparison. Albumin _t30_ (17.34 ± 4.94) vs. Albumin _t105_ (9.42 ± 1.42); Albumin _t120_ (8.74 ± 2.41); Albumin _t135_ (9.11 ± 2.46); p < 0.05 for all; intra group comparison].

### Evaluation of the tissue wet-to-dry weight ratio

The wet-to-dry-weight ratio (W/d) was evaluated before (W/d1) and after (W/d2) the perfusion with HES and Albumin, respectively. No statistically significant differences were detected between the HES perfusion group and the Albumin perfusion group at the start or end of perfusion (p > 0.05; Fig. 2B).

### Vascular perfusion with HES leads to a fluid shift to the luminal compartment

**Fig 3:**
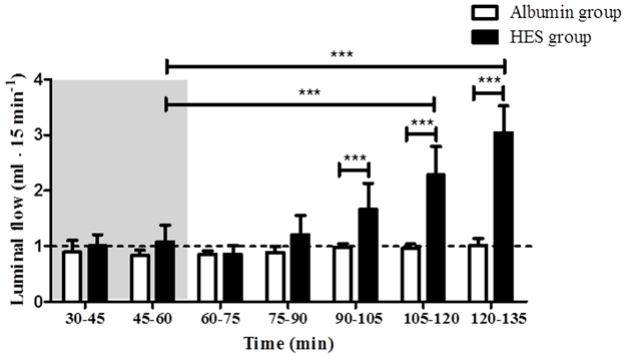
Luminal flow. Luminal effluent flow was measured in 15 minutes intervals. Areas shaded in grey indicate the equilibration phase. The default luminal flow rate is represented by a dashed line. Error bars denote the mean ± SD. Albumin (N = 7), HES (N = 7). ***, p < 0.001.

Vascular HES perfusion resulted in an alteration of fluid distribution and significantly increased luminal flow [HES _t45–60_ (1.08 ± 0.29 ml · 15 min^-1^) vs. HES _t105–120_ (2.28 ± 0.51 ml ·15 min^-1^); HES _t120–135_ (3.04 ± 0.48 ml · 15 min^-1^); p < 0.001 for all; intra group comparison; Fig. 3]. The luminal flow in the Albumin perfusion group showed no changes during perfusion (p > 0.05; intra group comparison; Fig. 3). The luminal flow between the Albumin perfusion group and the HES perfusion group differed significantly [Albumin _t90–105_ (0.98 ± 0,06 ml · 15 min^-1^); Albumin _t105–120_ (0.96 ± 0.08 ml · 15 min^-1^); Albumin _t120–135_ (1.01 ± 0.13 ml · 15 min^-1^) vs. HES _t90–105_ (1.66 ± 0.47 ml · 15 min^-1^); HES _t105–120_ (2.28 ± 0.51 ml · 15 min^-1^); HES _t120–135_ (3.04 ± 0.48 ml · 15 min^-1^); p < 0.001 for all; inter group comparison; Fig. 3]. Control experiments with prolonged HES application (without equilibration phase) showed that the luminal flow did not reach a saturation point and was still increasing after 135 minutes of HES perfusion (data not shown). In addition, measurements of the venous flow rate also suggested a significant loss of vascular fluid to the luminal compartment during perfusion with HES (S1 Fig.), whereas lymphatic flow was not different between the groups (data not shown).

### Vascular perfusion with HES increases the permeability of the endothelial and epithelial barrier

**Fig 4:**
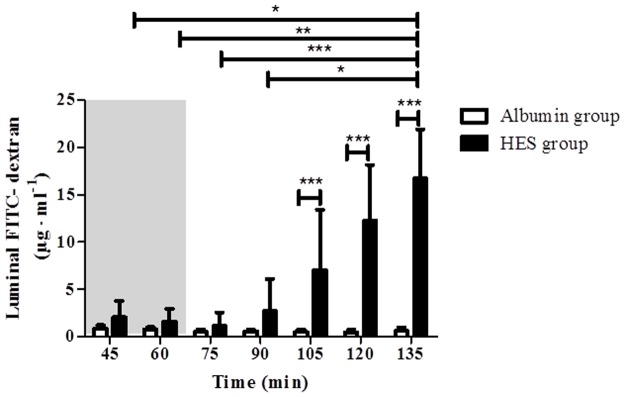
Luminal FITC-dextran concentrations. Luminal FITC-dextran concentrations were evaluated every 15 minutes and sufficient amounts of effluents for analyses were obtained at time point 45 min and beyond. Areas shaded in grey indicate the equilibration phase. Error bars denote the mean ± SD. Albumin (N = 7), HES (N = 7). *, p < 0.05; **, p < 0.01; ***, p < 0.001.

The endothelial and epithelial barrier integrity was estimated by determining the translocation of vascularly applied FITC-dextran into the luminal compartment. No changes in the luminal FITC-dextran concentration were detected in the Albumin perfusion group (p > 0.05; intra group comparison; Fig. 4). Vascular perfusion with HES time-dependently increased the luminal FITC-dextran concentration [HES _t45_ (2.04 ± 1.73 03BCg· ml^-1^); HES _t60_ (1.57 ± 1.37 μg· ml^-1^); HES _t75_ (1.14 ± 1.41 μg· ml^-1^); HES _t90_ (2.70 ± 3.39 μg· ml^-1^) vs. HES _t135_ (16.71 ± 5.17 μg · ml^-1^); p < 0.05 for t45 and t90, p < 0.01 for t60, p < 0.001 for t75; intra group comparison; Fig. 4]. Furthermore, compared to the Albumin perfusion group the luminal FITC-dextran concentration was significantly increased in the HES perfusion group [Albumin _t105_ (0.49 ± 0.26 μg· ml^-1^); Albumin _t120_ (0.47 ± 0.27 μg· ml^-1^); Albumin _t135_ (0.60 ± 0.37μg· ml^-1^) vs. HES _t105_ (7.03 ± 6.41 μg· ml^-1^); HES _t120_ (12.24 ± 5.91 μg· ml^-1^); HES _t135_ (16.71 ± 5.17 μg· ml^-1^); p < 0.001 for all; inter group comparison; Fig. 4]. Control experiments with prolonged HES application (without equilibration phase) showed that the luminal FITC-dextran concentration reached the saturation point after 105 minutes of HES perfusion (data not shown). In addition, measurements of the lymphatic FITC-dextran concentrations also showed a significant increase during perfusion with HES (S1 Fig.). Note: The differences in the FITC-dextran concentrations between the HES and Albumin group during the equilibration period (Fig. 4; HES/Albumin_t45_, HES/Albumin_t60_) can be explained by inter-individual variations of the intestines used in the respective perfusion experiments. The respective intestines were however not excluded from the study as all other physiologic and metabolic parameters were unaltered and within the expected limits.

### Vascular perfusion with HES alters the morphology of intestinal epithelial cells

**Fig 5:**
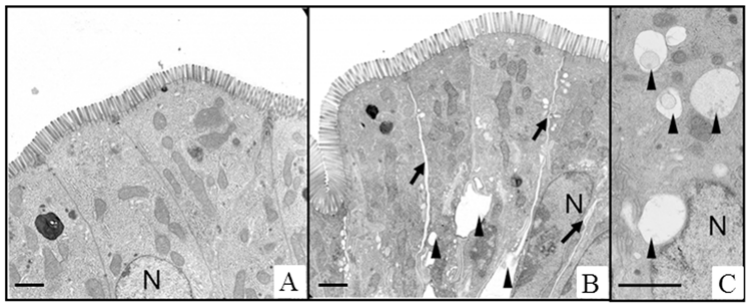
Ultrastructure of HES and Albumin perfused intestinal tissue. A) Albumin perfusion group, B and C) HES perfusion group. HES perfusion leads to widened intercellular spaces and appearance of intracellular vacuoles. Scale bars denote approximately 2μm. Black arrows, intercellular gaps; black arrowheads, vacuoles; N, nucleus.

The histomorphological damage of the intestinal epithelium was analysed by employing a histological stability score [14]. No significant differences were detected between the HES perfusion group and the Albumin perfusion group (HES 0.59 ± 0.09 vs. Albumin 0.61 ± 0.08; p > 0.05; data not shown). However, electron microscopic analyses suggested distinct cellular and subcellular changes in the small intestine after HES perfusion. In the HES perfusion group expanded intercellular spaces were found within the intestinal epithelium and epithelial cells contained numerous intracellular vacuoles. These changes were not observed after Albumin perfusion (Fig. 5).

### Vascular perfusion with HES impairs the resorptive capacity of the small intestine

**Fig 6:**
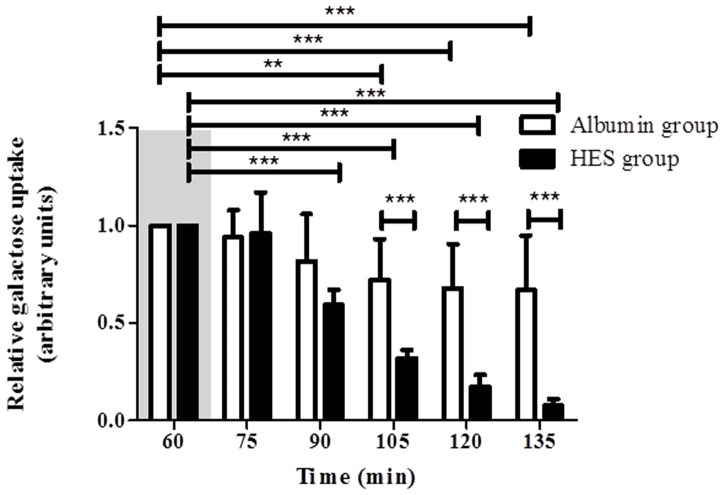
Intestinal galactose uptake. The relative galactose uptake (t_60_ = 1) was measured every 15 minutes. Areas shaded in grey indicate the equilibration phase. Error bars denote the mean ± SD. Albumin (N = 6), HES (N = 7). **, p < 0.01; ***, p < 0.001.

To validate the resorptive capacity and metabolic function of the isolated intestine during HES and Albumin perfusion, the luminal galactose uptake was measured. (LPR) Vascular perfusion with Albumin led to a time-dependent reduction of galactose uptake [Albumin _t60_ (set to 1) vs. Albumin _t105_ (0.72 ± 0.21); Albumin _t120_ (0.68 ± 0.23); Albumin _t135_ (0.67 ± 0.28); p < 0.01 for t105, p < 0.001 for t120 and t135; intra group comparison; Fig. 6]. A similar observation was found for the perfusion with HES which caused an even faster reduction of luminal galactose uptake [HES _t60_ (set to 1) vs. HES _t75_ (0.96 ± 0.21); HES _t90_ (0.59 ± 0.076); HES _t105_ (0.32 ± 0.04); HES _t120_ (0.17 ± 0.06); HES _t135_ (0.08 ± 0.03); p < 0.001 for all; intra group comparison; Fig. 6]. With increasing perfusion time, the galactose uptake differed significantly between the Albumin perfusion group and the HES perfusion group [Albumin _t105_ (0.72 ± 0.21); Albumin _t120_ (0.68 ± 0.23); Albumin _t135_ (0.67 ± 0.28) vs. HES _t105_ (0.32 ± 0.04); HES _t120_ (0.17 ± 0.06); HES _t135_ (0.08 ± 0.03); p < 0.001 for all; inter group comparison; Fig. 6].

## Discussion

The application of hydroxyethyl starch (HES) for volume resuscitation in critically ill patients is controversially discussed and several recent clinical studies have suggested adverse effects of HES substitution, leading to increased patient mortality [1, 3, 18]. Although of high clinical relevance, up to now the effects of HES on intestinal function and barrier integrity are mainly unknown.

### Ex-vivo model of the perfused intestine

Using a model of the isolated perfused mouse small intestine in combination with Krebs-Henseleit based perfusion media, we analysed the intestinal effects of a vascular perfusion with 3% HES in relation to a control perfusion with 3% Albumin. The employed system is a further development of our previously described model of the perfused rat intestine [14] and was designed to permit a detailed analysis of edema formation and permeability changes in the mouse intestine by monitoring fluid distributions and FITC-dextran transport from the vasculature to the interstitial space, the lymph and the lumen.

### Effects of HES on intestinal barrier function

One of our main results is a significantly altered fluid shift from the intestinal vasculature into the lumen of the small intestine during HES perfusion, indicative of an increased endothelial and epithelial barrier permeability. Our flow-rate data showed an increased luminal flow while the venous flow was reduced under perfusion with HES, suggesting a loss of vascular fluid into the intestinal lumen due to negative effects of HES on endothelial and epithelial barrier integrity. These results were confirmed by our FITC-dextran measurements, where FITC-dextran of 150 kDa was supplemented to the vascular perfusion buffer. While in the Albumin perfusion group only very low concentrations of FITC-dextran appeared in the luminal effluents, HES perfusion resulted in a dramatic increase of luminal FITC-dextran levels, confirming the hypothesis that HES impairs the endothelial and epithelial barrier function in the small intestine. Our results are also in accordance with data published by Gao et al. who employed a rat model of hemorrhagic shock and detected an initially higher intestinal bacterial translocation to the liver after HES resuscitation compared to Ringer’s solution [19]. A study by Li et al investigated the effects of different HES concentrations on ischemia-reperfusion injury of rat intestinal transplantations showing that high-concentration HES solutions (>3%) resulted in histomorphological damage and were not appropriate for intestinal preservation [20]. Employing an isolated perfused guinea pig heart model, Jacob et al. demonstrated a post-ischemic transient increase in vascular leak with Krebs-Henseleit buffer containing 6% HES 130/0.4 but not with 5% Albumin [21]. Interestingly, the authors also showed that augmenting the perfusion buffer with human Albumin improved the endothelial integrity after ischemia by protecting the endothelial glycocalyx [22]. In contrary to the above mentioned studies that suggest negative effects of HES on endothelial and epithelial barrier function, using a rat model of hemorrhagic shock Wang et al. demonstrated that HES resuscitation is able to reduce intestinal permeability [23] and that LPS- and sepsis induced capillary permeability is decreased after vascular HES perfusion [24, 25] pointing towards possible protective effects of a perfusion with HES under hemorrhagic and/or septic conditions, in which endothelial permeability may already be increased.

### Effects of HES on intestinal morphology

Although the clinical effects of a perfusion with HES are well described, the detailed mechanisms of HES on epithelial cells are still mainly unknown. Our electron microscopic experiments revealed numerous of intracellular vacuoles and enlarged intercellular spaces in the HES perfusion group, while these signs were absent after Albumin perfusion. Several authors detected similar structures in cells and classified them as HES containing vesicles that might be derived from lysosomes [26–28]. Others showed that the amount of vacuoles increased concomitantly with the dosage of HES used for perfusion [29]. Our findings of enlarged intercellular spaces after HES perfusion might be explained by an accumulation of interstitial fluids. HES molecules are able to overcome the endothelial barrier and reside in the interstitial compartment of the respective tissue, where they are stable for several years [28, 29]. As the HES molecule is osmotically highly active and is able to bind large amounts of water [30], the fluid shift from the vascular to the interstitial compartment might be increased, leading to the development of a transient interstitial edema. As a consequence of the increased tissue pressure, intercellular spaces in the intestinal epithelium expand and damage of tight junctions with increased fluid shift into the intestinal lumen may result. A similar morphological observation was also reported by Jacob et al. employing an isolated perfused heart model. Interstitial spaces within the cardiac tissue were more dense in the Albumin perfusion group than after perfusion with HES, pointing towards an interstitial fluid accumulation in the HES group [21].

### Effects of HES on the resorptive capacity of the intestine

In the intestine, the transfer of galactose across the luminal epithelium occurs by a sodium/galactose co-transporter (SGLT1), while the galactose transfer across the basolateral cell membrane is achieved by an uniporter (GLUT2) [31]. Our galactose uptake measurements in which 30 mM of lactose were supplied with the luminal perfusion buffer suggest several direct or indirect HES mediated mechanisms, which could function alone or in combination to impair galactose absorption in the intestine: (i) HES attenuates the digestion of lactose into galactose by inhibiting intestinal lactase activity and/or by reducing lactose concentration due to the increased fluid shift into the lumen (ii) HES impairs the uptake of galactose by intestinal epithelial cells via an interaction with SGLT1 (iii) HES negatively influences the transfer of galactose to the vascular compartment by inhibition of GLUT2. As we do not have sufficient biochemical data that support one the above mentioned mechanisms of HES action we cannot exclude the possibility that the attenuation of intestinal galactose uptake and metabolic function is rather and simply a consequence of the HES mediated deranged barrier integrity and disturbed tissue homeostasis than a direct effect of HES on the responsible transporter molecules and enzymes.

### Limitations of the study

The employed model of the isolated perfused small intestine offers the unique possibility to investigate organ-specific effects of a vascular perfusion with HES, while at the same time interfering systemical effects of HES are excluded. However, the following points should be considered when interpreting the results of our study. (I) A single pass application of a cell free vascular perfusate was performed in our study. Therefore, the model does not account for possible effects of systemic immune cells and humoral factors on the described HES and Albumin mediated mechanisms. (II) The perfusate used in our system was deficient in plasma proteins including coagulation factors, which may have an influence on the endothelial barrier function. (III) Possible effects mediated by the splanchnic nerves are not reflected in the perfused model of the small intestine, as the innervation was disrupted during organ preparation. However, the autonomously working enteric nervous system is still functioning as can be seen by the regular peristaltic movements of the perfused intestine in our model (S1 Movie).

### Conclusion

Taken together, we show that a vascular perfusion with clinically relevant concentrations of HES impairs the endothelial and epithelial barrier integrity as well as metabolic function of the small intestine. Our ex-vivo experiments extend recent studies that suggested negative effects of HES during fluid resuscitation and we propose the small intestine as a potential target for HES mediated adverse effects.

## Supporting Information

S1 Fig Relative venous flow. Venous flow was measured in 15 minutes intervals (t30–45 = 1). Areas shaded in grey indicate the equilibration phase. Bars denote the mean ± SD. Albumin (N = 7), HES (N = 7). **, p < 0.01; ***, p < 0.001.
(TIF)

S2 Fig FITC-dextran concentration. Lymphatic FITC-dextran concentrations were evaluated every 15 minutes. Areas shaded in grey indicate the equilibration phase. Bars denote the mean ± SD. Albumin (N = 7), HES (N = 7). *, p < 0.05.
(TIF)

S1 Movie Perfused small intestine. Real-time movie; scale bar = 2cm.
(MP4)
