# Correction: Hydroxyethyl Starch (HES 130/0.4) Impairs Intestinal Barrier Integrity and Metabolic Function: Findings from a Mouse Model of the Isolated Perfused Small Intestine

**DOI:** 10.1371/journal.pone.0127136

**Published:** 2015-04-23

**Authors:** The PLOS ONE Staff

## Notice of Republication

This article was republished on April 10, 2015, to correct figures that were distorted during the production process. The publisher apologizes for the error. Please download this article again to view the correct version. The originally published, uncorrected article and the republished, corrected article are provided here for reference.

## Supporting Information

S1 File Originally published, uncorrected article. (PDF)

S2 File Republished, corrected article. (PDF)
