# Supplementary figures and images for: Hydroxyethyl Starch (HES 130/0.4) Impairs Intestinal Barrier Integrity and Metabolic Function: Findings from a Mouse Model of the Isolated Perfused Small Intestine
Source: PLoS One. 2015 Mar 23;10(3):e0121497. doi: 10.1371/journal.pone.0121497 (PMC4370845; doi:10.1371/journal.pone.0121497)

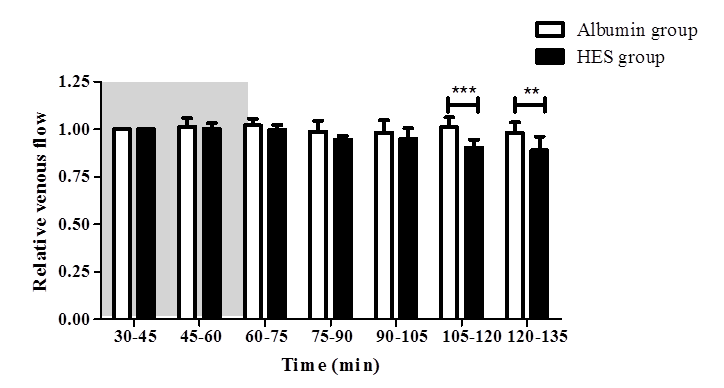

Supplement: S1 Fig — Venous flow was measured in 15 minutes intervals (t30–45 = 1). Areas shaded in grey indicate the equilibration phase. Bars denote the mean ± SD. Albumin (N = 7), HES (N = 7). **, p < 0.01; ***, p < 0.001. (TIF) [file pone.0121497.s001.tif]

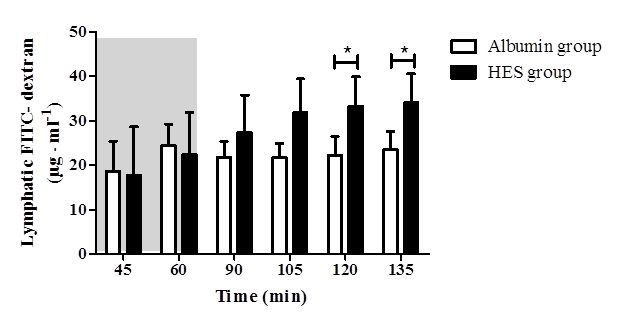

Supplement: S2 Fig — Lymphatic FITC-dextran concentrations were evaluated every 15 minutes. Areas shaded in grey indicate the equilibration phase. Bars denote the mean ± SD. Albumin (N = 7), HES (N = 7). *, p < 0.05. (TIF) [file pone.0121497.s002.tif]
